# Supplementary material for: Novel Driver Strength Index highlights important cancer genes in TCGA PanCanAtlas patients
Source: PeerJ. 2022 Aug 11;10:e13860. doi: 10.7717/peerj.13860 (PMC9375969; doi:10.7717/peerj.13860)
Supplement: Supplemental Information 2 [file peerj-10-13860-s002.zip › COHORTS/genes plots/detailed/2021_8_16_14_9_distribution_events_detailed_males_4.pdf]

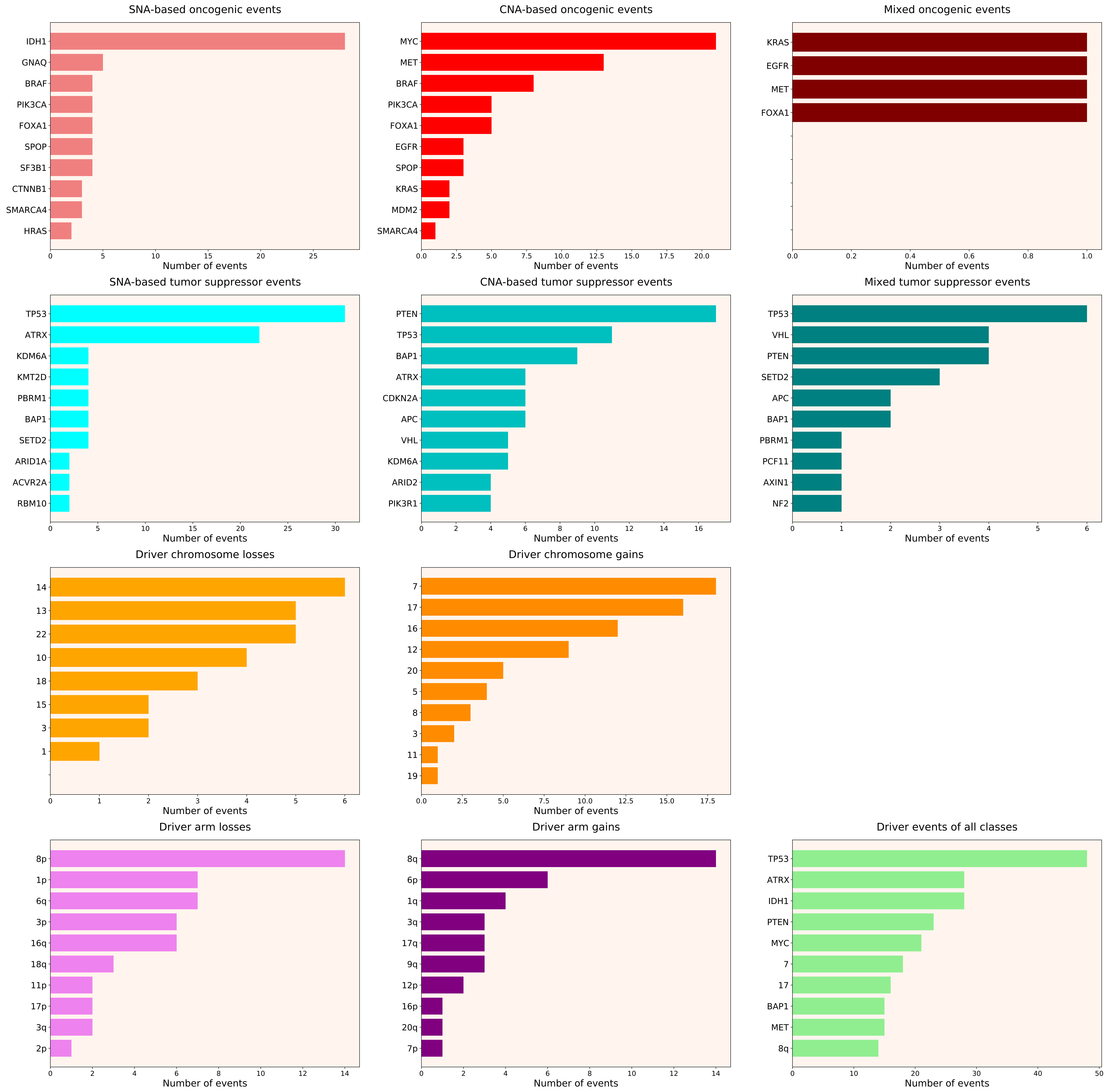
SNA-based oncogenic events
IDH1
GNAQ
BRAF
PIK3CA
FOXA1
SPOP
SF3B1
CTNNB1
SMARCA4
HRAS
0
5
10
15
20
25
Number of events
CNA-based oncogenic events
MYC
MET
BRAF
PIK3CA
FOXA1
EGFR
SPOP
KRAS
MDM2
SMARCA4
0.0
2.5
5.0
7.5
10.0
12.5
15.0
17.5
20.0
Number of events
Mixed oncogenic events
KRAS
EGFR
MET
FOXA1
0.0
0.2
0.4
0.6
0.8
1.0
Number of events
SNA-based tumor suppressor events
TP53
ATRX
KDM6A
KMT2D
PBRM1
BAP1
SETD2
ARID1A
ACVR2A
RBM10
0
5
10
15
20
25
30
Number of events
CNA-based tumor suppressor events
PTEN
TP53
BAP1
ATRX
CDKN2A
APC
VHL
KDM6A
ARID2
PIK3R1
0
2
4
6
8
10
12
14
16
Number of events
Mixed tumor suppressor events
TP53
VHL
PTEN
SETD2
APC
BAP1
PBRM1
PCF11
AXIN1
NF2
0
1
2
3
4
5
6
Number of events
Driver chromosome losses
14
13
22
10
18
15
3
1
0
1
2
3
4
5
6
Number of events
Driver chromosome gains
7
17
16
12
20
5
8
3
11
19
0.0
2.5
5.0
7.5
10.0
12.5
15.0
17.5
Number of events
Driver arm losses
8p
1p
6q
3p
16q
18q
11p
17p
3q
2p
0
2
4
6
8
10
12
14
Number of events
Driver arm gains
8q
6p
1q
3q
17q
9q
12p
16p
20q
7p
0
2
4
6
8
10
12
14
Number of events
Driver events of all classes
TP53
ATRX
IDH1
PTEN
MYC
7
17
BAP1
MET
8q
0
10
20
30
40
50
Number of events
